# Contribution of TMEM16F to pyroptotic cell death

**DOI:** 10.1038/s41419-018-0373-8

**Published:** 2018-02-20

**Authors:** Jiraporn Ousingsawat, Podchanart Wanitchakool, Rainer Schreiber, Karl Kunzelmann

**Affiliations:** Physiological institute, University of Regensburg, Universitätsstraße 31, D-93053 Regensburg, Germany

## Abstract

Pyroptosis is a highly inflammatory form of programmed cell death that is caused by infection with intracellular pathogens and activation of canonical or noncanonical inflammasomes. The purinergic receptor P2X_7_ is activated by the noncanonical inflammasome and contributes essentially to pyroptotic cell death. The Ca^2+^ activated phospholipid scramblase and ion channel TMEM16F has been shown earlier to control cellular effects downstream of purinergic P2X_7_ receptors that ultimately lead to cell death. As pyroptotic cell death is accompanied by an increases in intracellular Ca^2+^, we asked whether TMEM16F is activated during pyroptosis. The N-terminal cleavage product of gasdermin D (GD-N) is an executioner of pyroptosis by forming large plasma membrane pores. Expression of GD-N enhanced basal Ca^2+^ levels and induced cell death. We observed that GD-N induced cell death in HEK293 and HAP1 cells, which was depending on expression of endogenous TMEM16F. GD-N activated large whole cell currents that were suppressed by knockdown or inhibition of TMEM16F. The results suggest that whole cell currents induced by the pore forming domain of gasdermin-D, are at least in part due to activation of TMEM16F. Knockdown of other TMEM16 paralogues expressed in HAP1 cells suggest TMEM16F as a crucial element during pyroptosis and excluded a role of other TMEM16 proteins. Thus TMEM16F supports pyroptosis and other forms of inflammatory cell death such as ferroptosis. Its potent inhibition by tannic acid may be part of the anti-inflammatory effects of flavonoids.

## Introduction

Intracellular Ca^2+^ is enhanced during many biological processes including inflammation. Ca^2+^ mobilization is suggested to have a role in the regulation of NLRP3 (NOD, LRR, and pyrin domain-containing 3) inflammasome, a large supramolecular complex that activates caspase-1 during pyroptosis. Pyroptosis, a highly inflammatory form of programmed cell death, occurs upon infection with intracellular pathogens and is part of the antimicrobial response. In contrast to apoptosis, pyroptotic cell death results in plasma membrane (PM) rupture and release of so called damage-associated molecular pattern (DAMP) molecules^1^. Inflammasomes activate caspase-1 or caspase 11/4/5, which cleave the pore-forming N-terminal part of gasdermin D that drives the cell into lytic cell death^2–4^. Large gasdermin D pores are regarded as effectors of pyroptosis. These pores may lead to an increase in intracellular Ca^2+^ by permeabilizing the plasma membrane and probably also intracellular membranes. Moreover, noncanonical inflammasomes lead to caspase-11-dependent pyroptosis due to activation of pannexin-1, release of ATP binding to purinergic P2X_7_ receptors and consecutively increases intracellular Ca^2+^ ^5^. Notably the Ca^2+^ activated phospholipid scramblase and ion channel TMEM16F has been shown to participate in the cellular effects downstream of P2X_7_ receptors that finally lead to cell death^6^.

TMEM16F belongs to a family of 10 proteins (TMEM16A-K; anoctamin 1–10)^7^. These proteins are localized in the plasma membrane or in intracellular membrane compartments. Apart from TMEM16A and B, which are Ca^2+^ activated chloride channels without scrambling activity, other TMEM16 proteins expose phosphatidylserine to the outer plasma membrane leaflet and conduct ions when activated by an increase in intracellular Ca^2+^ ^8–14^. Evidence has been provided that TMEM16F (i) participates in cell shrinkage and presumably apoptotic cell death^15–17^, (ii) forms an outwardly rectifying Cl^−^ channel (ORCC) that is activated during death of immune cells^6,18,19^, and (iii) is activated during other forms of programmed cell death such as necroptosis and ferroptosis^20,21^. In the present study we asked whether TMEM16F is also activated during pyroptosis and, if so, whether it contributes to pyroptotic cell death.

## Results

### TMEM16F supports gasdermin D-induced cell death

**Fig. 1:**
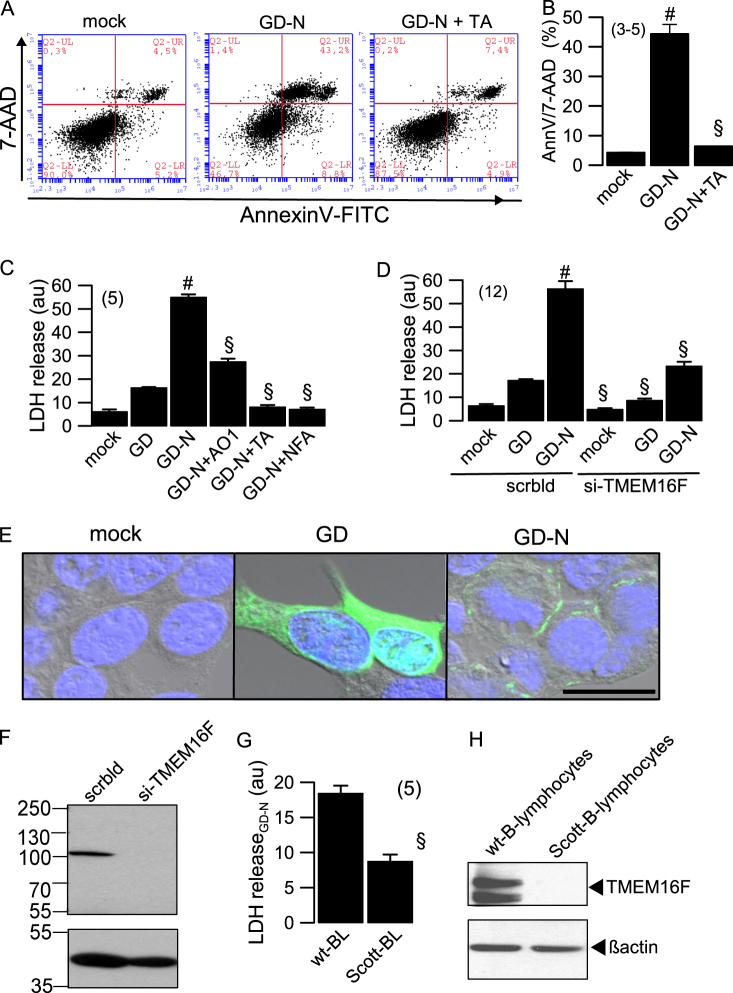
TMEM16F supports gasdermin D-induced cell death. **a**, **b** Dot blot diagram of cell death analysis by flow cytometry. Cell death (7-AAD and AnnexinV-FITC double staining) was significantly enhanced in HEK293 cells expressing the N-terminal pore–forming domain of gasdermin D (GD-N), but not in mock transfected cells. **c** LDH release in HEK293 cells transfected with empty plasmid (mock), full length gasdermin D (GD), or GD-N. Flow cytometry and LDH assays were performed 24 h after transfection. LDH release was inhibited by CaCCinhAO1 (AO1; 20 µM), tannic acid (TA; 10 µM) or niflumic acid (NFA; 100 µM). **d** LDH release measured in cells expressing empty plasmids, GD, or GD-N, which were treated with siRNA for TMEM16F or with scrambled RNA. **e** Immunocytochemistry of GD and GD-N expressed in HEK293 cells. **f** Western blot indicating knockdown of TMEM16F by siRNA. **g** LDH release in GD-N transfected human B-lymphocytes (wt-BL) and Scott-lymphocytes lacking expression of TMEM16F (Scott-BL). **h** Western blot of TMEM16F indicating lack of expression in Scott-B-lymphocytes. Mean ± SEM (number of FACS and LDH assays). ^#^significant increase when compared to mock (*p* < 0.05, ANOVA). ^§^significant inhibition by inhibitors or si-TMEM16F (*p* < 0.05, ANOVA)

In order to examine cell death induced by gasdermin D we expressed the amino-terminal pore–forming domain of gasdermin D (GD-N) in HEK293 cells. Cells were examined by flow cytometry after 24 h of expression, which indicated a high percentage of death, i.e., 7-AAD-positive cells, when compared to mock transfected cells (Fig. 1a, b). Interestingly, when GD-N-transfected cells were grown in the presence of the TMEM16F-inhibitor tannic acid (TA), the cell death-inducing effect of GD-N was completely abolished, suggesting that TMEM16F contributes to GD-N induced cell death. LDH-release was assessed after 24 h expression of full^−^length gasdermin (GD) and GD-N. While GD expressing cells showed only a small increase in LDH release, LDH release by GD-N expressing cells was remarkable, and was significantly inhibited by three different inhibitors of TMEM16F, CaCCinhAO1 (AO1), TA or niflumic acid (NFA) (Fig. 1c). Moreover, knockdown of TMEM16F, expressed endogenously in HEK293 cells, suppressed cell death induced by GD and GD-N (Fig. 1d, f). Expression of full^−^length gasdermin D (GD) and N-terminal fragment of gasdermin D (GD-N) was demonstrated by immunocytochemistry using gasdermin D antibody. While GD was found to be distributed homogenously throughout the cytosol, GD-N was localized as spots in the plasma membrane (Fig. 1e). Finally, GD-N induced LDH release was reduced in Scott B-lymphocytes (Scott-BL), which lack of expression of TMEM16F^19^, when compared to wt B-lymphocytes expressing TMEM16F (Scott-BL) (Fig. 1g, h). Taken together the data strongly suggest support of gasdermin D-induced cell death by TMEM16F.

### Increase in intracellular Ca2^+^ and activation of whole cell Cl^−^ currents by expression of pore forming gasdermin D

**Fig. 2:**
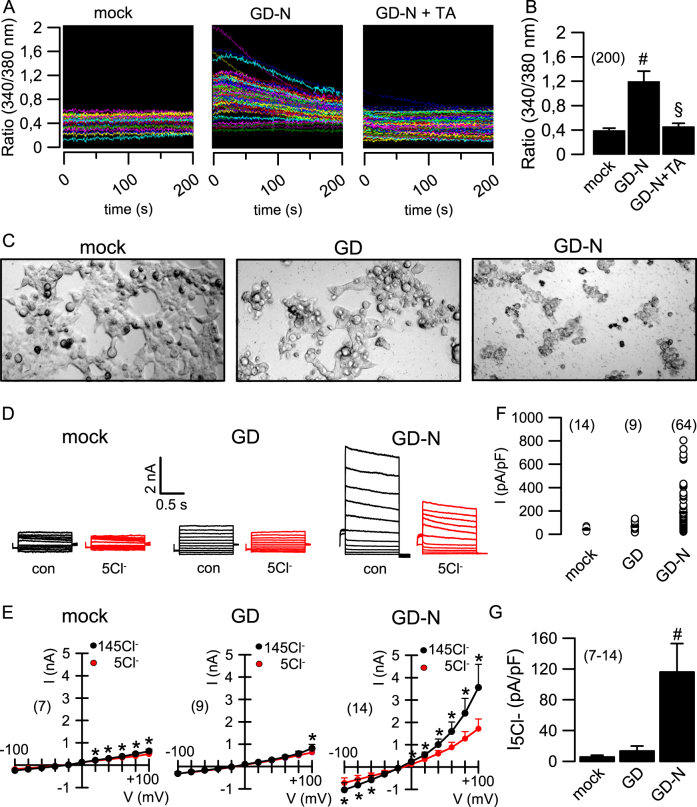
Increase of intracellular Ca^2+^ and activation of whole cell Cl^-^ currents by pore forming gasdermin D. **a**, **b** Original recordings from each 200 experiments (**a**) and summary of the baseline 340/380 fluorescence ratio in Fura-2 loaded cells expressing the N-terminal pore–forming domain of gasdermin D (GD-N) or empty plasmid (mock). Tannic acid (TA; 10 µM) inhibited the baseline Ca^2+^ increase in GD-N expressing cells. **c** Cell morphology of cells transfected with empty plasmid (mock), full length gasdermin D (GD), or GD-N. **d**,** e** Whole cell currents and corresponding current/voltage relationships obtained in non-stimulated mock-transfected HEK293 cells and cells expressing GD, or GD-N. The whole cell current in GD-N expressing cells was enhanced, which was inhibited by removal of extracellular chloride (5Cl^−^) from the bath solution. **f** Current densities for all individual cells examined by patch clamping, obtained at Vc = + 100 mV. **g** Summaries for the 5Cl^-^-inhibited whole cell current indicating activation of Cl^-^ permeable currents in cells expressing GD-N. Cells were voltage clamped ± 100 mV (1 s) in steps of 20 mV. Mean + /− SEM (number of experiments). ^#§^Significant difference when compared to mock or inhibition by tannic acid, respectively (unpaired *t*-test). *Significant inhibition by 5 Cl^−^ (*p* < 0.05; paired *t*-test)

**Fig. 3:**
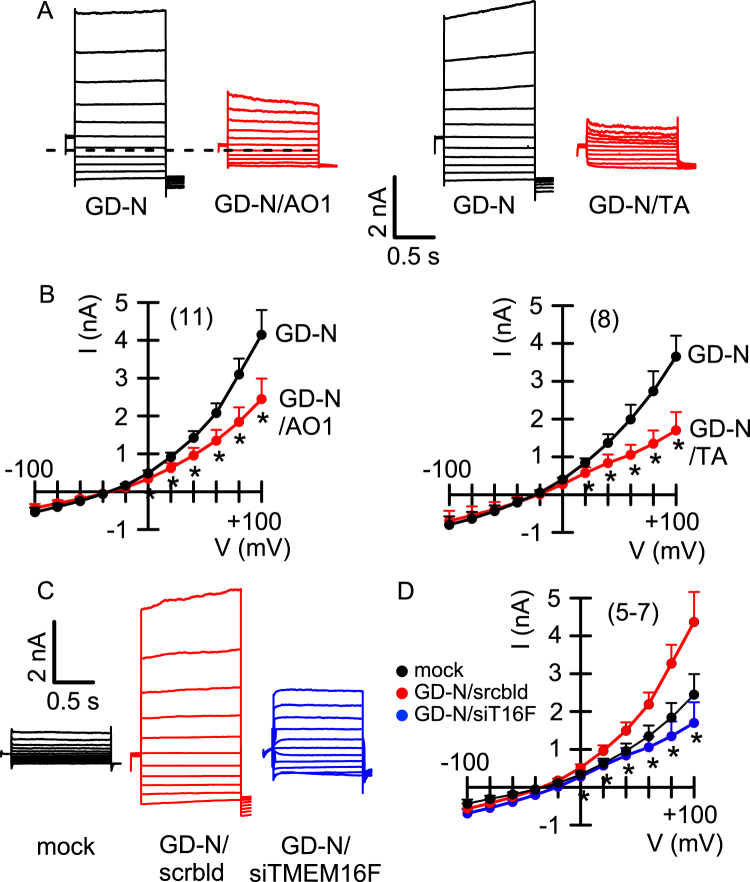
Gasdermin D-induced whole cell currents are inhibited by blockers of TMEM16F. **a** Whole cell currents obtained in non-stimulated HEK293 cells expressing the N-terminal pore-forming domain of gasdermin D (GD-N). The enhanced whole cell currents detected in these cells were significantly inhibited by CaCCinhAO1 (AO1; 20 µM) or tannic acid (TA; 10 µM). **b** Corresponding current/voltage relationships for the experiments shown in **a**. **c** whole cell currents measured in mock transfected HEK293 cells and cells expressing GD-N in the presence of scrambled RNA (scrbld) or after siRNA-knockdown of TMEM16F. **d** Corresponding current/voltage relationships. Cells were voltage clamped ± 100 mV (1 s) in steps of 20 mV. Mean±SEM (number of experiments). *Significant inhibition by AO1 and TA, respectively (*p* < 0.05; paired *t*-test)

As the N-terminal pore–forming domain of gasdermin D (GD-N) is likely to lead to an increase in intracellular Ca^2+^, we examined intracellular Ca^2+^ levels in cells expressing GD-N or in mock transfected cells, using the Ca^2+^ sensitive dye Fura2. The data indicate that baseline Ca^2+^ levels were enhanced in cells expressing GD-N. Interestingly, increase in intracellular Ca^2+^ in GD-N expressing cells was completely inhibited by tannic acid (Fig. 2a, b). This suggests that TMEM16 proteins, which are inhibited by tannic acid, have a role in GD-N induced Ca^2+^ increase. In fact, an earlier report demonstrated Ca^2+^ permeability of TMEM16F^14^. Because GD-N increased intracellular Ca^2+^, we examined ion currents activated during pyroptotic cell death (Fig. 2c, d). In whole cell patch clamp experiments we detected a whole cell current in HEK293 cells expressing GD-N, but not in cells expressing full length GD or in mock transfected cells (Fig. 2d, f). GD-N induced whole cell currents were outwardly rectifying and showed no consistent time dependence (Figs. 2d, e, 3). The enhanced whole cell currents found in GD-N expressing cells were potently inhibited by removal of extracellular Cl^−^, indicating a permeability of the current for Cl^−^ (Fig. 2e). The GD-N induced currents were also inhibited by the TMEM16F inhibitors CaCCinhAO1 (AO1) and tannic acid (TA; Fig. 3a, b). Moreover, knockdown of endogenous TMEM16F significantly inhibited GD-N induced whole cell currents (Fig. 3c, d). The results therefore suggest that whole cell currents induced by the pore forming domain of gasdermin-D are at least partially due to activation of TMEM16F.

### Knockdown of TMEM16F in HAP1 cells inhibits Ca^2+^-activated exposure of phosphatidylserine and GD-N induced cell death

**Fig. 4:**
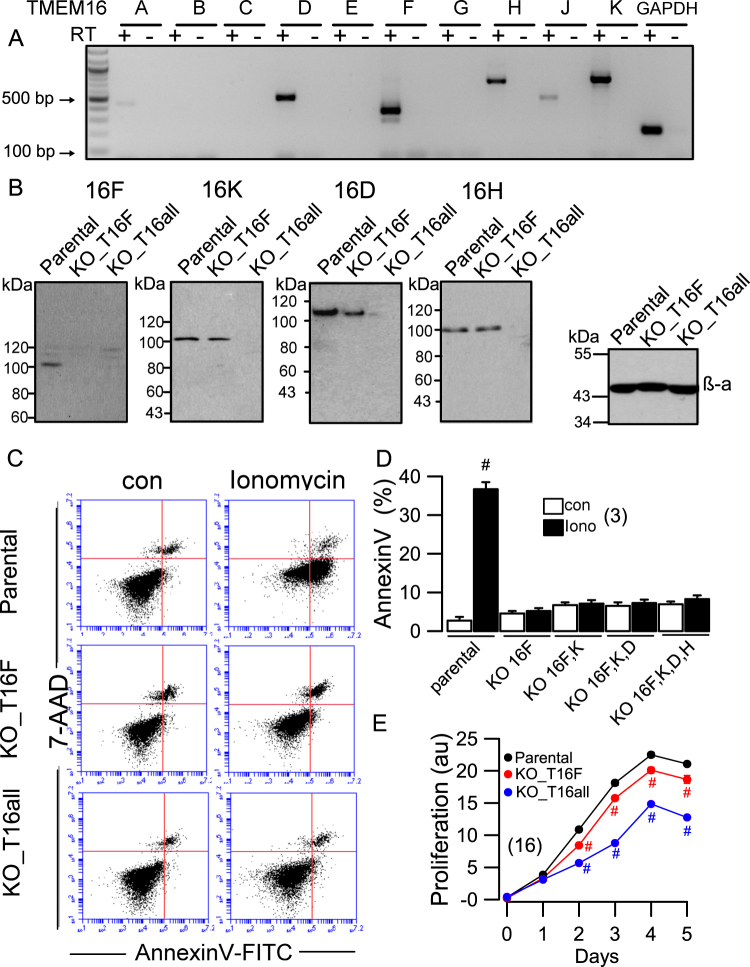
Knockdown of TMEM16F in HAP1 cells eliminates Ca^2+^-activated PS exposure. **a** RT-PCR analysis of HAP1 cells (horizon, Cambridge, UK) detected expression of TMEM16D,F,H,K. **b** Western blots indicating expression of TMEM16D,F,H,K in HAP1 parental cells (Parental) and knockout of expression of TMEM16F (KO_16F) or knockout of TMEM16D,F,H,K (KO_T16all) by gene editing. Very right panel shows loading controls in Parental, KO_16F, and KO-all (β-actin). **c**, **d** Dot blot diagram from flow cytometry and summary of annexin V positive cells, indicating Ca^2+^-dependent activation of phospholipid scrambling (exposure of phosphatidylserine, PS) by ionomycin in parental HAP1 cells but not in cells lacking expression of TMEM16F (KO_16F) or TMEM16F and additional TMEM16 proteins. **e** Proliferation of HAP1 cells lacking expression of TMEM16F or lacking expression of all endogenous TMEM16 proteins was significantly reduced when compared with parental cells. Mean±SEM (number of experiments). ^#^Significant activation by ionomycin, or inhibition of proliferation (*p* < 0.05; unpaired *t*-test)

We examined TMEM16F and its role for gasdermin-induced cell death in the haploid leukemia cells line HAP1^22^. This cell line was chosen because it was also available as TMEM16F-knockout cell line (horizon, Cambridge, UK). Moreover, it was straightforward to knockout additional TMEM16 paralogues expressed in HAP1 cells by CRISPR/Cas9, and to examine their potential contribution to gasdermin-induced cell death. RT-PCR analysis indicated expression of TMEM16D,F,H,K in HAP1 parental cells (Fig. 4a). Expression of these TMEM16 paralogues in HAP1 parental cells was confirmed by Western blotting (Fig. 4b). No expression of TMEM16F was detected in TMEM16F-knockout HAP1 cells (KO-T16F). Moreover, CRISPR/Cas9 knockout of the remaining TMEM16 paralogues D,H,K (KO-T16all) was also confirmed by Western blotting (Fig. 4b). TMEM16A was not detected in HAP1 cells. FACS analysis demonstrated Ca^2+^-induced exposure of phosphatidylserine (phospholipid scrambling) in parental cells, which was absent in KO-T16F and KO-T16all cells (Fig. 4c, d). Knockout of TMEM16F or TMEM116D,F,H,K progressively attenuated cell proliferation (Fig. 4e). An anti-proliferative effect of TMEM16F knockout has also been observed in another study^23^. Moreover, similar to the study by Schenk et al, we observed slightly enhanced phospholipid scrambling after knockdown of TMEM16F and other TMEM16 proteins (Fig. 4d). The scramblase Xkr8^24^ was also found to be expressed in HAP1 cells (in semiquantitative (sq) RT-PCR and Western blots, not shown). Interestingly, by sqRT-PCR we found an upregulation of Xkr8 expression relative to GAPDH from 0.49 ± 0.031 (parental) to 0.86 ± 0.058 (KO_16F) and 1.1 ± 0,37 (KO_T16all; all *n* = 3). This may explain the lower basal scrambling activity in TMEM16F expressing cells. This was further supported by experiments in which we knocked down expression of Xkr8 by siRNA (by 95.1 %, parental; 96.2%, KO_T16F; 66.9%, KO_T16all), which significantly reduced basal scrambling in the three different cell lines. Finally, we reported earlier that TMEM16 proteins have a significant impact on cytosolic Ca^2+^ levels, which may affect cell proliferation and basal scrambling activity^25^. In fact, knockdown of TMEM16K had a pronounced inhibitory effect on TNFα-induced cell death^26^.

**Fig. 5:**
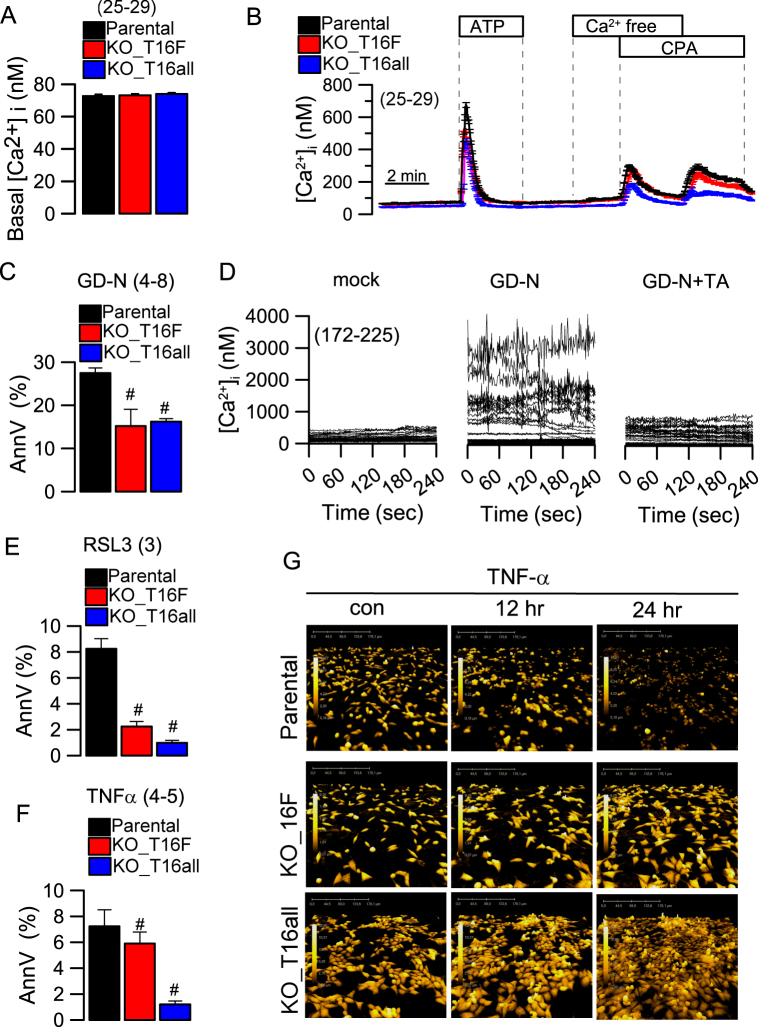
Knockdown of TMEM16F attenuates different cell death pathways. **a** Summary of basal intracellular Ca^2+^ levels, which was not affected by knockout of TMEM16 proteins. **b** Increase in intracellular Ca^2+^ levels by stimulation with ATP (100 µM) or cyclopiazonic acid (CPA, 100 µM) in the presence of a Ca^2+^ free extracellular bath solution. Increase in intracellular Ca^2+^ by ATP was significantly reduced in KO_T16F and KO_T16all cells. **c** Summary of cell death (7-AAD and annexinV-FITC double staining) induced by expression of GD-N in parental, KO-T16F, and KO_T16all cells. **d** Baseline Ca^2+^ levels measured in mock transfected cells and cells expressing GD-N. Increase in intracellular Ca^2+^ by expression of GD-N was completely inhibited by tannic acid (TA; 10 µM). **e**,** f** Summary of cell death induced by ferroptosis (incubation with 1 µM RSL3 and 10 µM erastin for 24 h) or apoptosis (incubation with 100 ng/ml TNFα for 24 h). **g** Cell morphology assessed by quantitative holographic phase microscopy. Scale bar indicates cell height. Mean + /− SEM (number of cells). ^#^Significant inhibition when compared to parental cells (*p* < 0.05; unpaired *t*-test)

Knockout of TMEM16F or all TMEM16 proteins did not affect basal Ca^2+^ levels (Fig. 5a). We examined enhanced intracellular Ca^2+^ concentrations by stimulation with ATP (100 µM), or cyclopiazonic acid (CPA, 10 µM) in the presence of a Ca^2+^ free extracellular bath solution. ATP-induced rise in intracellular Ca^2+^ was significantly reduced in KO_T16F and KO_T16all cells, corresponding to earlier observations in other cell types^25^ (Fig. 5b). Using HAP1 cells, we examined the role of TMEM16F for gasdermin-induced cell death. Cell death induced by expression of GD-N was twice as frequent in parental cells when compared to cells lacking expression of TMEM16F (KO-T16F). Additional knockdown of TMEM16D,H,K did (KO_T16all) did not further reduce GD-N induced cell death (Fig. 5c). Single additional knockdown of TMEM16D,H,K did not show additional effects. Similar to GD-N transfected HEK293 cells, we also observed enhanced baseline Ca^2+^ levels in GD-N expressing HAP1 cells, which was potently suppressed by tannic acid (Fig. 5d). We also examined other forms of programmed cell death, such as ferroptosis (using the cystine import inhibitor erastin and the GPX4 inhibitor RSL3) or apoptosis (by incubation with TNFα). Both ferroptosis and apoptosis were significantly attenuated in cells lacking expression of TMEM16F (Fig. 5e, f). Analysis of cell morphology by quantitative holographic phase microscopy demonstrated the impressive attenuation of apoptosis in cells lacking expression of TMEM16F (Fig. 5g). These results are in line with earlier observations that suggested an impact of TMEM16F on apoptotic and ferroptotic cell death, respectively, and an activation of TMEM16F during necroptosis^6,15,16,20,21^. The present report now adds pyroptosis to this list, as another regulated cell death pathway that includes simultaneous activation of TMEM16F. Future studies should examine whether TMEM16F could serve as a drug target to control both inflammatory and non-inflammatory cell death.

## Discussion

Increase in intracellular Ca^2+^ by initial Ca^2+^ store release and subsequent store operated Ca^2+^ entry (SOCE) is essential to drive a number of processes that operate in immune cells, such as degranulation and cytotoxicity by CD8^+^T cells and natural killer (NK) cells, production of reactive oxygen species in neutrophils via NADPH oxidase, and phagocytosis by macrophages^27^. We demonstrated earlier that Ca^2+^ activated TMEM16F mediates a number of functions in macrophages, downstream of P2X_7_ receptors^6^. Initial cell shrinkage and subsequent cell swelling was related to progressive activation of TMEM16F. Gasdermin D binds to phosphatidylinositol species and phosphatidylserine and forms large nonselective pores^4^. Expression of TMEM16F is particularly high in macrophages, where it supports phagocytic activity and cell death. The present data now imply that TMEM16F may have also a significant impact on pyroptotic cell lysis and subsequent phagocytosis by neutrophils. Consequently, TMEM16F is likely to affected inflammation. Notably, TMEM16F was found recently to be activated during ferroptotic cell death, induced by lipid peroxidation^21,28^. Taken together, TMEM16F contributes to different forms of regulated cell death such as apoptosis, ferroptosis and pyroptosis. It may therefore contribute to inflammation, hypersensitivity and generation of pain, and might represent a pharmacological target during inflammatory diseases”.

Although it is emerging that TMEM16F and probably other TMEM16 paralogues have a role in programmed cell death, it is not clear whether they do so by operating as a channel/scramblase or by affecting intracellular Ca^2+^ levels^6,15,16,29^. In fact, recent work suggests that TMEM16F and other TMEM16 proteins control intracellular Ca^2+^ signals, thereby affecting numerous cellular functions^25,30^. TMEM16F may operate as a plasma membrane localized nonselective Ca^2+^ influx channel^14^ or as a passive Cl^−^/nonselective channel that counterbalances charge movements occurring during ER Ca^2+^ store release or Ca^2+^ influx^25^.

Inflammatory stimuli that enhance intracellular Ca^2+^ levels or modify plasma membrane phospholipids also activate TMEM16F^15,21,28^. Thus PLA_2_ not only acts as a central switch that turns on synthesis of numerous proinflammatory mediators^31^, but also serves as a powerful activator of TMEM16F^15^. Similarly, proinflammatory and lipid peroxidizing reactive oxygen species (ROS) are also powerful activators, and both PLA_2_ and ROS do not require increase in cytosolic Ca^2+^ to activate TMEM16F^15,21^. In contrast, TMEM16F did not contribute to necroptotic cell death, although it might be activated during necroptosis^20^. In contrast, the present data suggest a significant contribution of TMEM16  to pyroptosis. In this context is might be of interest that membrane stabilizing tannic acid and other flavonoids^32^ are potent inhibitors of TMEM16F and strong anti-inflammatory/ antioxidant molecules^15,33–35^. Remarkably, tannic acid completely suppressed ATP-induced membrane blebbing in mouse macrophages^6^. Finally, TMEM16F is essential for activation of ADAM17 (Disintegrin and Metalloproteinase 17), which in turn is required for release of interleukin 6 receptors causing proinflammatory IL-6 trans-signaling^36,37^. The present data expand the proinflammatory role of TMEM16F to another highly inflammatory form of programmed cell death.

## Methods

### Generation of TMEM16-knockout cell lines by Crispr/Cas9, RT-PCR, and cell culture

HAP1 cells were transfected with the CRISPR_CD4 plasmids obtained through ThermoFisher Scientific® (Schwerte, Germany) using Lipofectamine3000 (Life technologies, Darmstadt, Germany) according to the manufacturer’s instructions (Table 1). Cells were collected and selected 3 days after transfection. Selected cells were expanded and genomic DNA was isolated to determine the efficiency of locus-specific double-strand break formation using GeneArt® Genomic Cleavage Detection (Invitrogen). HAP1 cells (Thermo Fisher scientific) were grown in Iscove’s modified Dulbecco’s medium (IMDM) supplemented with 10% fetal bovine serum at 37 °C in 5% CO_2_. Total RNA was isolated from CRISPR cells using RNeasy Mini-Kit (Qiagen; Hilden, Germany). Two μg of total RNA was reverse transcribed in 50 μl buffer for 1 h at 40 °C, using a random primer and M-MLV reverse transcriptase (Promega, Mannheim, Germany). Thirty cycles of RT–PCR was performed using standard procedures (GoTaq DNA Polymerase, Promega), 1 μl RT using primers for TMEM16 proteins described earlier^38^ (Table 2). PCR products were analyzed on 2% agarose gels.

**Table 1:** CRISPR_CD4 plasmids (Life technologies)

Target Name	Sequence (5′ to 3′)
CRISPR_CD4_ANO4.1	TGACGGGCCAAAGAGTGATG
CRISPR_CD4_ANO4.2	GTAGTATTTGCATATCCAGC
CRISPR_CD4_ANO4.3	AGAGAATCGTCATCTTTGCA
CRISPR_CD4_ANO8.1	CCGATGACCACACGCTGCTA
CRISPR_CD4_ANO8.2	ATACGTGGCGGTGACAAAGA
CRISPR_CD4_ANO8.3	AGCACGTCGCAGTTCTCTGT
CRISPR_CD4_ANO10.1	GGTGGTCATAGAACTTGCTC
CRISPR_CD4_ANO10.2	GCAAGTTCTATGACCACCAA

**Table 2:** Primers used for RT-PCR of TMEM16A-K (ANO1-ANO10)

Anoctamin	PCR-Primer	Size (bp)
ANO1 (16a)	5‘-CGACTACGTGTACATTTTCCG 5‘-GATTCCGATGTCTTTGGCTC	445
ANO2 (16b)	5‘-GTCTCAAGATGCCAGGTCCC 5‘-CTGCCTCCTGCTTTGATCTC	553
ANO3 (16c)	5‘-CTTCCCTCTTCCAGTCAAC 5‘-AAACATGATATCGGGGCTTG	461
ANO4 (16d)	5‘-CGGAAGATTTACAGGACACCC 5-GATAACAGAGAGAATTCCAATGC	503
ANO5 (16e)	5‘-GAATGGGACCTGGTGGAC 5‘-GAGTTTGTCCGAGCTTTTCG	713
ANO6 (16 f)	5‘-CAAATGGAGGAGGAGGAGGAC 5‘-GGTGCGTGTACTTTTACAAATAC	356
ANO7 (16 g)	5‘-CTCGGGAGTGACAACCAGG 5‘-CAAAGTGGGCACATCTCGAAG	470
ANO8 (16 h)	5‘-GGAGGACCAG CCAATCATC 5‘-TCCATGTCATTGAGCCAG	705
ANO9 (16j)	5‘-GCAGCCAGTT GATGAAATC 5‘-GCTGCGTAGGTAGGAGTGC	472
ANO10 (16k)	5‘-GTGAAGAGGAAGGTGCAGG 5‘-GCCACTGCGAAACTGAGAAG	769
GAPDH	5‘-GTATTGGGCGCCTGGTCAC 5‘-CTCCTGGAAGATGGTGATGG	200 bp

### Cell proliferation assay

Cells were plated in 96-well plates at a density of 8 × 10^3^ cells per well for the time duration as indicated (0, 24, 48, 72, 96 and 120 h). At the end of incubation, cells were washed with 150 μl of phosphate buffered solution (D-PBS^Ca2+/Mg2+^). Afterwards cells were incubated for 2 h in 100 μl of fresh media containing 0.5 mg/ml of the tetrazolium salt MTT. The dark blue formazan product was dissolved with DMSO and measured the absorbance at 540 nm.

### Western Blotting of TMEM16D,F,H,K and immunocytochemistry

Cells were collected and lysed in 1% NP40 lysis buffer containing 1×protease inhibitor cocktail and DTT. Protein (30–50 µg) was separated by 8.5% SDS-PAGE and transferred to nitrocellulose membranes. Membranes were blocked with 5% NFM/TBST or 5% NFM/PBST at RT for 1 h and were incubated overnight at 4 °C with goat polyclonal anti-TMEM16D (diluted 1:500 in 3% NFM/PBST, Santa Cruz), rabbit polyclonal anti-TMEM16F (diluted 1:5000 in 5% NFM/TBST, Thermo Fisher Scientific, USA), goat polyclonal anti-TMEM16H (diluted 1:2000 in 5% NFM/PBST, Santa Cruz), rabbit polyclonal anti-TMEM16K (diluted 1:2000 in 5% NFM/PBST, ANOVA, USA), or rabbit polyclonal anti-actin (diluted 1:10,000 in 5% NFM/TBST, Sigma). Subsequently, membranes were incubated with secondary AB at RT for 2 h. Immunoreactive signals were visualized using supersignal chemiluminescence substrate detection kit (Pierce Biotechonology, Rockford, USA). Gasdermin D full length and N-terminal fragment were labeled using gasdermin antibody sc-376318 (Santa Cruz, Heidelberg, Germany) and a secondary anti-mouse FITC-conjugated antibody.

### Volume measurements using HoloMonitor^TM^

Cells were seeded in 35 mm dishes at a density of 300,000 cells/ dish. Cell morphology was observed after application of 10 µM ABT737 in IMDM medium containing 10% FBS or 100 ng/ml TNFα in OptiMEM for 24 h in a cell culture incubator (37 °C, humidified air, 5% CO_2_). The cell volume was calculated by quantitative phase microscopy in the HoloMonitor^TM^ time-lapse cytometer (Phase Holographic imaging PHI, Lund, Sweden).

### Flow cytometry

Cells were grown in 24-well plates for 24 h and were incubated afterwards with 10 μM ABT737 in IMDM medium containing 10% FBS or 100 ng/ml TNFα in OptimMEM medium for 24 h. Floating cells were collected initially, whereas adherent cells were collected by treatment with Accutase and centrifuged 500 × *g* 4 °C for 10 min. Cell pellets were washed with cold DPBS and stained with FITC-labeled annexin V and 7-AAD for 10 min at room temperature in the dark. At least 10,000 cells were analyzed immediately with BD Accuri™ C6 flow cytometer.

### Caspase assay

Cells were plated in 24-well plates for 24 h and then incubated with 10 μM ABT737 in IMDM medium containing 10% FBS or 100 ng/ml TNFα in OptiMEM for 24 h. Floating cells were collected initially, whereas adherent cells were collected by treatment with Accutase and centrifuged 500 × *g* 4 °C for 10 min. Cell pellets were washed with cold DPBS and stained with 5 μM DEVD-NucView488 substrate for 30 min at room temperature in the dark. At least 10,000 cells were analyzed immediately with a BD Accuri™ C6 flow cytometer.

### Lactate dehydrogenase release

HEK293 cells (8 × 10^4^ cells/ml) were transfected with 2 ng/µl plasmid (pcDNA 3.1 (mock), full^−^length gasdermin D (GD), or the amino-terminal pore–forming domain of gasdermin D (GD-N), using Lipofectamine 2000. Supernatants were collected and measured using the CytoTox96^®^ non-radioactive cytotoxicity assay (Promega) at a wavelength of 490 nm. Percentage of LDH release was calculated as 100 × (experimental LDH-spontaneous LDH) / (maximum LDH release-spontaneous LDH).

### Ca^2+^ measurements

Cells were seeded on glass coverslips and loaded with 2 μM Fura-2/AM and 0.02% Pluronic F-127 (Life Technologies, Germany) in ringer solution (mmol l^−1^: NaCl 145; KH_2_PO_4_ 0,4; K_2_HPO_4_ 1,6; Glucose 5; MgCl_2_ 1; Ca^2+^-Gluconat 1,3) for 1 h at room temperature. Fluorescence ratios at 340/380 nm were detected at 37 °C using an inverted microscope IMT-2 (Olympus, Nuremberg, Germany) and a high-speed polychromator system (Visi-Chrome, Puchheim, Germany). After calibration, intracellular Ca^2+^ concentrations upon background subtraction were calculated. Alternatively, cells were transfected with pcDNA3.1 or the Ca^2+^ sensor Pl^−^G-CaMP2 for 3 days. ER Ca^2+^ signals were detected as previously described^25^.

### Patch clamping

Cells were grown on coated glass cover slips. Patch pipettes were filled with a cytosolic-like (standard) solution containing KCl 30, K -gluconate 95, NaH_2_PO_4_ 1.2, Na_2_HPO_4_ 4.8, EGTA 1, Ca -gluconate 0.758, MgCl_2_ 1.03, D-glucose 5, ATP 3, pH 7.2. In all experiments the bath was perfused for at least 5 min with control Ringer solution after establishing the whole cell configuration, in order to assure stable recording conditions. The intracellular (pipette) Ca^2+^ activity was 0.1 µM. In some experiments, all except of 5 mM Cl^−^ was replaced by impermeable gluconate (5Cl^−^). Fast whole cell current recordings were performed as described recently^18^. In brief, the bath was perfused continuously with Ringer solution (containing (in mM) 145 NaCl, 0.4 KH2PO4, 1.6 K2HPO, 4.6 D-glucose, 1 MgCl2 1.3 Ca^2+^ gluconate; pH 7.4) at a rate of 8 ml/min. Patch pipettes had an input resistance of 2–4 MΩ and measured whole cell currents were corrected for serial resistance. Currents were recorded using a patch clamp amplifier (EPC 7, List Medical Electronics, Darmstadt, Germany), the LIH1600 interface and PULSE software (HEKA, Lambrecht, Germany) as well as Chart software (AD Instruments, Spechbach, Germany). In regular intervals, membrane voltage (*V*c) was clamped in steps of 20 mV from −100 to +100 mV from a holding voltage of −100 mV. Current densities at +100 mV clamp voltage were calculated by dividing the measured whole cell currents by cell capacitance.

### Materials and statistical analysis

All compounds used were of highest available grade of purity and were from Sigma-Aldrich (Germany), Tocris Bioscience (Bristol, United Kingdom), or Merck (Darmstadt, Germany). Cell culture reagents were from Invitrogen and Capricorn Scientific (Ebsdorfergrund, Germany). FACS reagents were from BD biosciences and BioLegend GmbH (Koblenz, Germany). Data are presented as Mean±SEM. Student’s *t*-test for paired or unpaired samples or Fisher’s exact test and analysis of variance were used, for statistical analysis. *P* < 0.05 was accepted as significant difference.
